# Danger-associated molecular pattern molecules take unexpectedly a central stage in Nlrp3 inflammasome–caspase-1-mediated trafficking of hematopoietic stem/progenitor cells

**DOI:** 10.1038/s41375-021-01158-9

**Published:** 2021-02-23

**Authors:** Arjun Thapa, Mateusz Adamiak, Kamila Bujko, Janina Ratajczak, Ahmed K. Abdel-Latif, Magda Kucia, Mariusz Z. Ratajczak

**Affiliations:** 1 Stem Cell Institute at James Graham Brown Cancer Center, University of Louisville, Louisville, KY USA; 2 Center for Preclinical Studies and Technology, Department of Regenerative Medicine at Medical University of Warsaw, Warsaw, Poland; 3 Division of Cardiovascular Medicine, Gill Heart Institute, University of Kentucky, Lexington, KY USA

**Keywords:** Haematopoietic stem cells, Stem cells, Haematopoietic system

## Abstract

Like their homing after transplantation to bone marrow (BM), the mobilization of hematopoietic stem/progenitor cells (HSPCs) is still not fully understood, and several overlapping pathways are involved. Several years ago our group proposed that sterile inflammation in the BM microenvironment induced by pro-mobilizing agents is a driving force in this process. In favor of our proposal, both complement cascade (ComC)-deficient and Nlrp3 inflammasome-deficient mice are poor G-CSF and AMD3100 mobilizers. It is also known that the Nlrp3 inflammasome mediates its effects by activating caspase-1, which is responsible for proteolytic activation of interleukin-1β (IL-1β) and interleukin-18 (IL-18) and their release from cells along with several danger-associated molecular pattern molecules (DAMPs). We observed in the past that IL-1β and IL-18 independently promote mobilization of HSPCs. In the current work we demonstrated that caspase-1-KO mice are poor mobilizers, and, to our surprise, administration of IL-1β or IL-18, as in the case of Nlrp3-KO animals, does not correct this defect. Moreover, neither Caspase-1-KO nor Nlrp3-KO mice properly activated the ComC to execute the mobilization process. Interestingly, mobilization in these animals and activation of the ComC were both restored after injection of the DAMP cocktail eATP+HGMB1+S100A9, the components of which are normally released from cells in an Nlrp3 inflammasome–caspase-1-dependent manner. In addition, we report that caspase-1-deficient HSPCs show a decrease in migration in response to BM homing factors and engraft more poorly after transplantation. These results for the first time identify caspase-1 as an orchestrator of HSPC trafficking.

## Introduction

Research on enhancing egress of hematopoietic stem/progenitor cells (HSPCs) from bone marrow (BM) into peripheral blood (PB) by administration of pro-mobilizing drugs seeks to optimize the regimens now available in the clinic to harvest enough cells for transplantation purposes. One has to take into consideration (i) that some patients mobilize poorly, (ii) the overall costs involved in this procedure, and (iii) HSPC donor convenience [1–3]. In our previous work we demonstrated that the distal part of the complement cascade (ComC) is important for optimal mobilization of HSPCs. Mice deficient in the fifth component of complement (C5) are poor mobilizers [4, 5], and weak activation of C5 correlates with poor mobilization in patients [4, 6]. Based on these findings, we became interested in the role of other elements of innate immunity that are involved in this process.

Recently, we discovered that the Nlrp3 inflammasome, which is expressed in innate immunity cells and HSPCs, plays an important role in the egress of HSPCs from BM into PB. We reported that Nlrp3 inflammasome-deficient mice are poor mobilizers in response to standard drugs employed in the clinic, such as cytokine granulocyte colony-stimulating factor (G-CSF) and the CXCR4 receptor blocking agent and partial agonist [7–9]. The Nlrp3 inflammasome protein complex is located in the cytoplasm of innate immunity cells and, as we demonstrated recently, also in HSPCs in an inactive form. Upon activation, the complex becomes a functional protein aggregate composed of several Nlrp3 molecules (speck complexes) containing Nlrp3 proteins, ASC, and pro-caspase-1 [10–14]. In response to Nlrp3 activation, pro-caspase-1 becomes active caspase-1 and cleaves several proteins into their mature forms, thereby activating them inside cells. The important targets are pro-interleukin-1β (IL-1β) and pro-interleukin-18 (IL-18), which after cleavage are released from cells as potent proinflammatory mediators [10–12, 14]. Activated caspase-1 also cleaves gasdermin-D protein [15], which is an effector protein for forming pores in cell membranes. Gasdermin-D pores are also important in the release from cells of several danger-associated molecular patterns (DAMPs), such as extracellular adenosine triphosphate (eATP), high mobility group box 1 (HMGB1) protein, and S100 calcium-binding protein A9 (S100A9) [16–21], which are directly or indirectly involved in activation of the ComC [4, 5, 16, 21].

It is known that, as an important mediator involved in innate immunity responses, the Nlrp3 inflammasome plays pleotropic roles in regulating the biology of cells. If properly activated, it promotes cell trafficking; however, if hyperactivated, it may perturb the cell’s osmotic potential, leading to cell swelling and a lytic, highly inflammatory form of programmed cell death known as pyroptosis [10, 11, 15, 22, 23]. In our previous work we demonstrated that injection of the Nlrp3 inflammasome-activation mediators IL-1β or IL-18 into mice induces mobilization of functional HSPCs [7–9, 24]. Based on the fact that the Nlrp3 inflammasome activates caspase-1 to release these cytokines from cells, we became interested in whether caspase-1 deficiency would result in poor mobilization. Here we report that, indeed, mice that are caspase-1 deficient (caspase-1 KO) are poor mobilizers. To our surprise, however, as is the case for Nlrp3-KO mice, the poor mobilization status in caspase-1-KO mice is not reversed by administration of recombinant IL-1β or IL-18.

To address this unexpected observation, we focused on the role of the Nlrp3 inflammasome–caspase-1 axis in the release of DAMPs, which are activators of the ComC that are required for optimal mobilization of HSPCs. We found that poor mobilization status in caspase-1-KO and Nlrp3- KO mice is restored to normal status after injection of DAMPs, which are correlated with enhanced activation of the ComC. Thus, our results further support important roles for innate immunity, BM sterile inflammation in response to pro-mobilizing agents, and the involvement of Nlrp3 inflammasome-released DAMPs, which activate the ComC to ensure optimal egress of HSPCs from BM into PB. Moreover, we also report that caspase-1-deficient HSPCs show a decrease in migration in response to BM homing factors and engraft more poorly after transplantation. Overall, for the first time our results identify caspase-1 as an orchestrator of HSPC trafficking.

## Materials and methods

### Animals

Pathogen-free, 6–8-week-old female C57BL/6J wild-type (WT), B6.129S6-Nlrp3^tm1Bhk^/J (Nlrp3-KO), and B6N.129S2-Casp1tm1Flv/J (Caspase-1-KO) mice were purchased from the Central Laboratory for Experimental Animals, Medical University of Warsaw or the Jackson Laboratory (Bar Harbor, ME, USA) at least 2 weeks before experiments. Animal studies were approved by the Animal Care and Use Committee of the Warsaw Medical University (Warsaw, Poland) and the Institutional Animal Care and Use Committee of the University of Louisville (Louisville, KY, USA).

### In vitro mobilization studies

Mice were mobilized with G-CSF (180 μg/kg per day; Amgen, Thousand Oaks, CA, USA) for 4 days by subcutaneous injection or AMD3100 (5 mg/kg; Sigma-Aldrich, St. Louis, MO, USA) for 1 day via intraperitoneal injection (IP). Depending on the experimental setup, mice were also injected once with IL-1α or IL-33 (1 μg per mouse) or a mixture of IL-1β and IL-18 (0.5 μg each per mouse; Sino Biological, Beijing) via IP. In some experiments, mice were injected with a mixture of interleukins IL-1β and IL-18 (0.5 μg each per mouse) and/or a DAMP molecule cocktail (ATP [3 mg/kg; Sigma-Aldrich, St. Louis, MO, USA] + HMGB1 [1.5 μg; Sino Biological, Beijing] + S100A9 [2 μg; Sino Biological, Beijing]) per mouse in the presence of AMD3100 or G-CSF. The DAMP cocktail was injected each day for 3 days for the G-CSF mobilization group and once for the AMD3100 mobilization group. In a separate experiment, mice were also injected with the caspase-1 inhibitor VX765 [25] (40 mg/kg; AdooQ Biosciences, Irvine, CA, USA) in the presence of AMD3100 or G-CSF by IP. VX765 was injected once every day for 6 days.

The mice receiving interleukin(s), VX765, or a cocktail of interleukins and DAMPs in the presence of G-CSF were bled from the retro-orbital plexus (6 h after the last injection), and blood samples were drawn for plasma and hematology analysis. PB from these mice was obtained from the vena cava (with a 25-gauge needle and 1-ml syringe containing 250 U heparin). The blood samples from AMD3100-administered groups of animals were collected 1 h after injection(s). PB mononuclear cells (PBMNCs) were obtained by hypotonic lysis of red blood cells (RBCs) in BD Pharm Lyse buffer (BD Biosciences, San Jose, CA, USA) as described before [4, 7].

### Murine bone marrow-derived mononuclear cells (BMMNCs)

Cells were obtained by flushing experimental mouse tibias and femurs. RBCs were lysed with BD Pharm Lyse buffer (BD Biosciences, San Jose, CA, USA), washed, and resuspended in appropriate media [4, 7].

### Evaluation of HSPC mobilization

For evaluation of circulating colony-forming unit-granulocyte/macrophage (CFU-GM) and SKL cells, the following formulas were used: (number of white blood cells [WBCs] × number of CFU-GM colonies)/number of WBCs plated = number of CFU-GM per µl of PB; and (number of WBCs × number of SKL cells)/number of gated WBCs = number of SKL cells per μl of PB [4, 7].

### PB parameter counts

To obtain WBC counts, 50 μl of PB was taken from the retro-orbital plexus of mice into microvette EDTA-coated tubes (Sarstedt Inc., Newton, NC, USA) and run on a HemaVet 950FS hematology analyzer (Drew Scientific Inc., Oxford, CT, USA) within 2 h of collection [4, 7].

### Clonogenic CFU-GM and BFU-E assays

PBMNCs were resuspended in methylcellulose base medium (R&D Systems, Minneapolis, MN, USA). The medium for clonogenic CFU-GM assays was supplemented with 25 ng/ml recombinant murine granulocyte/macrophage colony-stimulating factor (mGM-CSF) and 10 ng/ml recombinant murine interleukin 3 (mIL-3). To perform burst-forming unit-erythroid (BFU-E) assays, BMMNC samples were suspended in methylcellulose supplemented with erythropoietin (5 U/ml) and IL-3 (10 ng/ml; PeproTech, Rocky Hill, NJ, USA). Cells were incubated for 7 days (37 °C, 95% humidity, and 5% CO_2_) for CFU-GM and 7 days for BFU-E assays. The CFU-GM and BFU-E colonies were scored using a simple inverted microscope (Olympus, Center Valley, PA, USA) [4, 7, 26].

### Flow cytometry analysis

For the staining of Lin^–^/Sca-1^+^/c-Kit^+^ (SKL) cells, the following monoclonal antibodies were used: FITC–anti-CD117 (also known as c-Kit, clone 2B8; BioLegend, San Diego, CA, USA), PE–Cy5–anti-mouse Ly-6 A/E (also known as Sca-1, clone D7; eBioscience, San Diego, CA, USA), and anti-mouse lineage-marker antibodies, including anti-CD45R (also known as B220, clone RA3-6B2), anti-Ter-119 (clone TER-119), anti-CD11b (clone M1/70), anti-T cell receptor β (clone H57-597), anti-Gr-1 (clone RB6-8C5), and anti-TCRγδ (clone GL3) conjugated with PE (BD Biosciences, San Jose, CA, USA). Staining was performed in RPMI-1640 medium containing 2% FBS. All monoclonal antibodies were added at saturating concentrations, and the cells were incubated for 30 min on ice, washed twice, and analyzed using an LSRII flow cytometer (BD Biosciences, San Jose, CA, USA) [4, 7, 26].

### Transwell migration assay

RPMI-1640 medium containing 0.5% BSA was used for migration-assay experiments. A 650-μl volume of medium with or without stromal-derived factor 1 (SDF-1, 5 ng/ml), sphingosine-1-phosphate (S1P, 0.1 μM), ATP (10 μM), antimicrobial peptide cathelicidin bioactive fragment LL-37 (2.5 μg/ml), or a mixture of SDF-1 and LL-37 was added to the lower chamber of a Costar Transwell 24-well plate (Corning Inc., Corning, NY, USA). An aliquot (1 × 10^6^ cells per 100 μl) of experimental mouse BMMNC suspension was loaded onto the upper chamber containing 5-μm pore filters and then incubated for 3 h at 37 °C in a 5% CO_2_ incubator. Following incubation, an aliquot of cells from the lower chamber was harvested and resuspended in human methylcellulose base medium (R&D Systems, Minneapolis, MN, USA) supplemented with murine GM-CSF (25 ng/ml) and IL-3 (10 ng/ml). Next, the cultures were incubated for 7 days at 37 °C in a 5% CO_2_ incubator. The CFU-GM colonies were then counted under an inverted microscope [24, 26, 27].

### Short-term homing experiments

WT mice were irradiated with a lethal dose of γ-irradiation (10 Gy). Twenty-four hours later, the animals were transplanted by injecting PKH67 green fluorescent dye (Sigma-Aldrich, St Louis, MO, USA)-labeled BMMNCs (5 × 10^6^/100 µl) from WT or Casp1-KO mice via tail vein injection. Twenty-four hours after transplantation, BMMNCs from the femurs were isolated by Ficoll–Paque density-gradient centrifugation. The cells were divided into two aliquots, one of which was analyzed by flow cytometer for PKH67-positive cells, while the other was resuspended in human methylcellulose base medium supplemented with murine GM-CSF (25 ng/ml) and IL-3 (10 ng/ml). The cultures were incubated for 7 days at 37 °C in a 5% CO_2_ incubator. The CFU-GM colonies were then counted using a simple inverted microscope [24, 26].

### Evaluation of engraftment

For engraftment experiments, WT mice were irradiated with a lethal dose of γ-irradiation (10 Gy). Twenty-four hours after irradiation, the animals were transplanted with 1.5 × 10^5^ BM cells from WT or Casp1-KO mice via tail vein injection. Twelve days after transplantation, the femora of transplanted mice were flushed with phosphate-buffered saline (PBS). BMMNCs were isolated by Ficoll–Paque density-gradient centrifugation. The cells were then plated in serum-free human methylcellulose base medium supplemented with mGM-CSF (25 ng/ml) and IL-3 (10 ng/ml). After 7 days of incubation (37 °C, 95% humidity, and 5% CO_2_), the CFU-GM colonies were scored under an inverted microscope. Spleens from the transplanted mice were also removed and fixed in Telesyniczky’s solution, and the CFU-S colonies on the surface of the spleen were counted [24, 26].

### Recovery of leukocytes and platelets

WT mice were irradiated with a lethal dose of γ-irradiation (10 Gy). Twenty-four hours later, the irradiated animals were transplanted with 7.5 × 10^5^ BM cells from WT or Casp1-KO mice via tail vein injection. At the time intervals indicated, the transplanted mice were bled from the retro-orbital plexus to obtain WBC and platelet (PLT) counts, as described earlier [24, 26, 27]. Briefly, 50 μl of PB was drawn into EDTA-coated Microvette tubes (Sarstedt Inc., Newton, NC, USA) and the samples run on a HemaVet 950FS hematology analyzer within 2 h of collection (Drew Scientific Inc., Oxford, CT, USA) [24, 26, 27].

### qRT-PCR analysis of Nlrp3 inflammasome complex gene expression

BMMNCs from WT or Casp1-KO experimental mice injected with PBS, AMD3100, or G-CSF were harvested, and the total RNA was isolated with the RNeasy Mini kit (Qiagen Inc., Germany), and the purified RNA was reverse transcribed with iScript reverse transcriptase (Biorad, Hercules, CA, USA). Quantitative evaluation of the target genes was then performed using SYBR Green PCR Master Mix reagents (Applied Biosystems, Carlsbad, CA, USA) and specific primers. The samples were run on an ABI Prism 7500 sequence detection system (Applied Biosystems, Carlsbad, CA, USA). The PCR cycling conditions were 95 °C (15 s), 40 cycles at 95 °C (15 s), and 60 °C (1 min). According to melting point analysis, only one PCR product was amplified under these conditions. The relative quantity of a target gene, normalized to the β2-microglobulin gene as the endogenous control and relative to a calibrator, was expressed as 2^–ΔΔCt^ (fold difference). The following primer pairs were used for analysis:

*mIL-1β*

forward primer: 5′-TCACAGCAGCACATCAACAA-3′

reverse primer: 5′-TGTCCTCATCCTGGAAGGTC-3′

*mIL-18*

forward primer: 5′-ACAACTTTGGCCGACTTCAC-3′

reverse primer: 5′-GGGTTCACTGGCACTTTGAT-3′

*mAIM2*

forward primer: 5′-AAAACTGCTCTGCTGCCTCT-3′

reverse primer: 5′-GATGGCTTCCTGTTCTGCCA-3′

*mNLRP1*

forward primer: 5′-GCTGAATGACCTGGGTGATGGT-3′

reverse primer: 5′-CTTGGTCACTGAGAGATGCCTG-3′

*mNLRP3*

forward primer: 5′-GCTGCTGAAGATGACGAGTG-3′

reverse primer: 5′-TTTCTCGGGCGGGTAATCTT-3′

*mASC*

forward primer: 5′-GCCAGAACAGGCACTTTGTG-3′

reverse primer: 5′-AGTCAGCACACTGCCATGC-3′

*mHMGB1*

forward primer: 5′-TAAAAAGCCCAGAGGCAAAA-3′

reverse primer: 5′-GCSGCSATGGTCTTCCACCT-3′

*mS100A9*

forward primer: 5′-TGGTGGAAGCACAGTTGG-3′

reverse primer: 5′-CATCAGCATCATACACTCCTCAA-3′

*mβ2M*

forward primer: 5′-ATGCTATCCAGAAAACCCCTCAAAT-3

reverse primer: 5′-AACTGTGTTACGTAGCAGTTCAGTA-3′

### Caspase-Glo® 1 inflammasome assay

To measure caspase-1 activity, the caspase-Glo® 1 inflammasome assay kit (Promega, Madison, WI, USA) was used. Briefly, murine tibias and femurs from WT mice were flushed, and BMMNCs were obtained after lysis of the RBCs using 1 × BD Pharm Lyse buffer (BD Pharmingen, San Jose, CA, USA). The cells were then suspended in RPMI medium containing 0.5% BSA (0.2 × 10^6^/50 µl) and plated in a 96-well plate in the absence or presence of ATP (10 µM), S1P (0.1 µM), or SDF-1 (100 ng/ml). The plates were incubated at 37 °C in 5% CO_2_ for 3 h. To measure caspase-1 activity directly in cells, reconstituted Caspase-Glo® reagent, which contains a luminogenic caspase-1 substrate, Z-WEHD-aminoluciferin, was added to the samples. The samples were mixed gently and incubated at room temperature for 60 min. The luminescence was measured using a plate-reading luminometer (Beckman Coulter TDX 8000, Brea, CA, USA) at 60, 90, and 120 min after adding the reagents.

### Enzyme-linked immunosorbent assay

BMMNCs from WT or Casp1-KO or NLRP3 KO mice injected with the mixture of interleukins or interleukins and DAMPs in the presence or absence of AMD3100 or G-CSF were isolated as described above. The residual RBCs were lysed using Lyse buffer (BD Biosciences, San Jose, CA, USA). Then, the samples were incubated in 0.5% BSA containing RPMI for 24 h at 37 °C in a 5% CO2 incubator. The culture supernatant was collected. A 100 µl of the conditioned medium samples were used in this experiment [24, 28]. The experiments were performed in triplicates using complement C5a mouse ELISA kit (Abcam, Cambridge, MA, USA) as described in manufacturer’s protocol.

### Statistical analysis

All results are presented as mean ± SD. Statistical analysis of the data was done using Student’s *t*-test for unpaired samples, with *p* ≤ 0.05 considered significant.

## Results

### Effect of the IL-1 cytokine family on HSPC mobilization

**Fig. 1:**
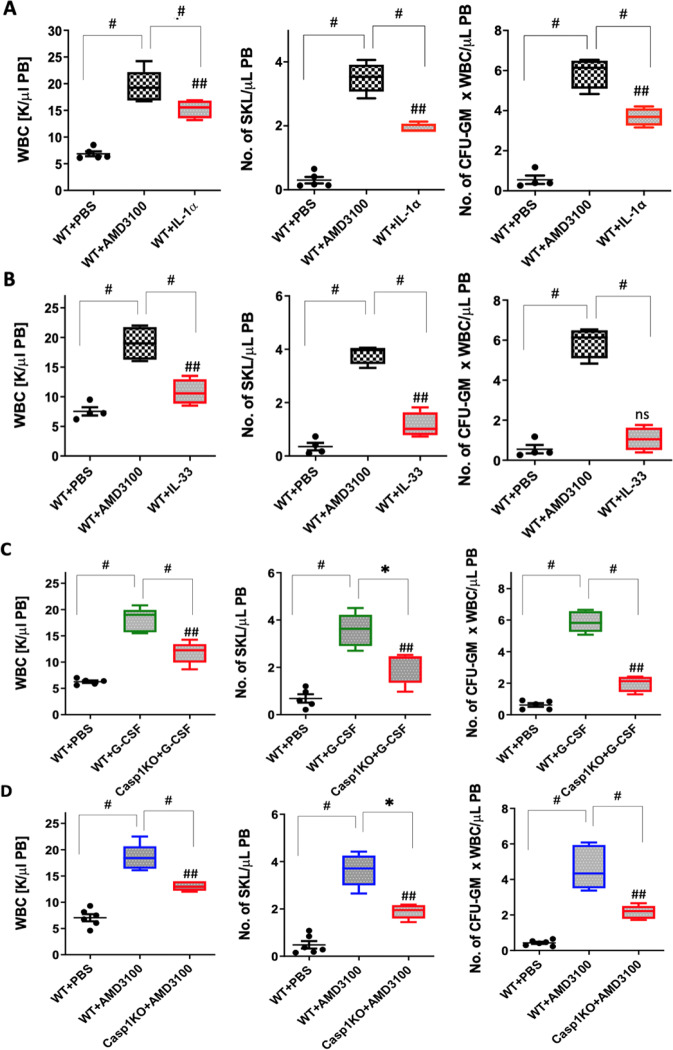
G-CSF and AMD3100 mediated mobilization experiments in normal mice in response to IL-1α and IL-33 and poor G-CSF and AMD3100 mobilization in caspase-1-KO animals. **A** WT mice were injected with PBS, AMD3100 (5 mg/kg), or IL-1α (1 µg/mice), and peripheral blood (PB) parameters for WBCs (left), SKL cells (middle), and CFU-GM clonogenic progenitors (right) were analyzed. **B** WT mice were injected with PBS, AMD3100 (5 mg/kg), or IL-33 (1 µg/mice), and PB parameters for WBCs (left), SKL cells (middle), and CFU-GM clonogenic progenitors (right) were analyzed. AMD3100, IL-1α, and IL-33 were injected once. AMD3100-injected mice were sacrificed 1 h post injection, whereas the mice that had received IL-1α or IL-33 were sacrificed 6 h post injection. To perform a CFU-GM colony assay, peripheral blood mononuclear cell (PBMNC) samples were cultured in human methylcellulose medium supplemented with growth factors. The PBMNCs were stained with appropriate antibodies, and the SKL cell population was analyzed using a flow cytometer. The number of SKL cells mobilized into PB was calculated using the formula: WBCs × SKL cells/gated WBCs = SKL cells/μl. The number of CFU-GM/μl in PB was evaluated by the formula: [WBCs] × CFU-GM colonies/WBCs plated. The data are presented as means ± SE, and an unpaired Student’s *t*-test was used for the determination of significance (*p ≤ 0.05, ^#^*p* ≤ 0.005, and ^##^*p* ≤ 0.005 or ^ns^ no significant WT+PBS vs WT+IL-1α). **C** Caspase-1-KO mice are poor G-CSF and AMD3100 mobilizers. WT mice were injected with PBS or G-CSF (180 µg/kg), while caspase-1-KO (Casp1-KO) mice were injected with G-CSF (180 µg/kg), and the PB parameter profiles for WBCs (left), SKL cells (middle), and CFU-GM clonogenic progenitors (right) were analyzed. PBMNC samples were cultured for CFU-GM colonies, and stained PBMNCs were analyzed by flow cytometer for SKL cell analysis. **D** WT mice were injected with PBS or AMD3100 (5 mg/kg), while Casp1-KO mice were injected with AMD3100 (5 mg/kg), and the PB parameter profiles for WBCs (left), SKL cells (middle), and CFU-GM clonogenic progenitors (right) were analyzed. AMD3100 was injected once, whereas G-CSF was given on 4 consecutive days. AMD3100-injected mice were sacrificed 1 h post injection, whereas G-CSF-injected animals were sacrificed 6 h after the last injection. The numbers of SKL cells and CFU-GM progenitors mobilized into PB were calculated using the formula described in Materials and methods. The data are presented as means ± SE, and an unpaired Student’s *t*-test was used for the determination of significance (*p ≤ 0.05, ^#^*p* ≤ 0.005, and ^##^*p* ≤ 0.005 WT+PBS vs Casp1-KO+G-CSF).

In our previous work we presented evidence that administration of IL-1β to normal WT mice induces effective mobilization of HSPCs [7–9]. We also demonstrated that a similar effect was obtained with another cytokine, IL-18, which, like IL-1β, is released in a caspase-1-dependent manner in response to activated Nlrp3 inflammasomes [7–9]. Here we repeated mobilization experiments in WT animals and employed two other members of the IL-1 interleukin family, IL-1α and IL-33 [29–31]. As shown in Fig. 1A, B, IL-1α, but not IL-33, promoted egress of HSPCs from BM into PB. This demonstrates that the pro-mobilization effect is restricted to specific members of the IL-1 cytokine family, namely, IL-1β and IL-1α. In particular, IL-1β and IL-18 are products of activation of the Nlrp3 inflammasome, which we have demonstrated in the past as playing an important role in the trafficking of HSPCs [7–9, 24]. Therefore, we focused on the role of these cytokines, which are released in an Nlrp3 inflammasome–caspase-1-dependent manner.

### Caspase-1 KO mice are poor G-CSF and AMD3100 mobilizers

Since both IL-1β and IL-18 are released from cells after cleavage of their pro-forms by caspase-1, in order to learn more about the role of caspase-1 in the mobilization process, we mobilized WT mice with G-CSF and AMD3100 in the presence of the caspase-1 inhibitor VX765 [25]. As expected, we found that these animals mobilized poorly (Supplementary Fig. 1). Next, to confirm this result we employed caspase-1-KO animals and mobilized them with G-CSF or AMD3100 (Fig. 1C, D). Again, the number of mobilized WBC, SKL cells, and clonogenic progenitors circulating in PB was reduced in caspase-1-KO animals compared with normal WT mouse controls.

We also evaluated the number of SKL cells and CFU-GM and BFU-E clonogenic progenitors in the BM of caspase-1-KO mice under steady-state conditions and found that these animals have ~10–12% higher numbers in BM (Supplementary Fig. 2). Since progenitor cells are released continuously from BM and circulate in PB, this finding may indicate that, under steady-state conditions, egress of these cells from BM is also somewhat impaired. This question, however, requires further study.

### BMMNCs from caspase-1-KO mice show less-efficient migration in response to SDF-1, S1P, and eATP gradients and display worse homing and engraftment properties than cells from normal control animals

**Fig. 2:**
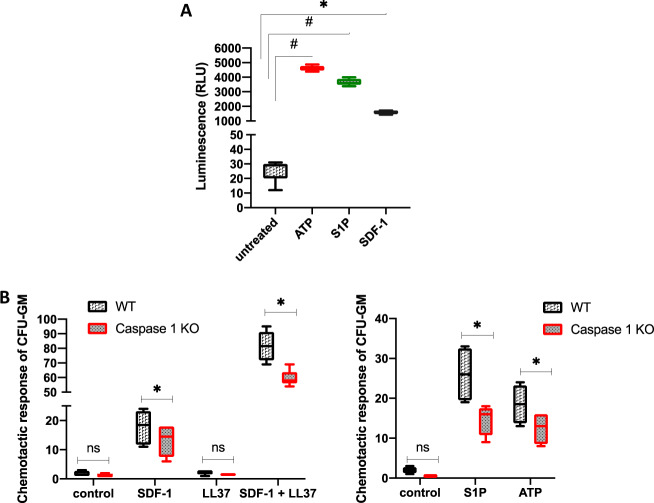
Caspase-1 is activated in BMMNCs in response to BM chemoattractants, and caspase-1-KO BMMNCs show a decrease in chemotaxis. **A** luminescence measured in WT BMMNCs cultured in the absence or presence of ATP (10 µM), S1P (0.1 µM), or SDF (100 ng/ml) for 3 h at 37 °C prior to adding Caspase-1 Glo® 1 reagent. **B** The number of CFU-GM colonies formed from WT or Casp1-KO BMMNC samples recovered from the lower chamber of a 24-well Costar Transwell plate into which four different sets of chemoattractants, including SDF-1 (5 ng/ml), S1P (0.1 μM), ATP (0.25 μg/ml), LL-37 (2.5 μg/ml), or SDF-1 plus LL-37, were added to chemoattract the BMMNCs placed on the upper layer of the cell culture insert through a permeable membrane. The data are presented as means ± SE. Unpaired Student’s *t*-test was used for the determination of significance (*p ≤ 0.05 and ^#^*p* ≤ 0.005).

Trafficking of HSPCs is regulated by selected chemotactic factors, such as SDF-1, S1P, and eATP [20, 24, 32–35]. Therefore, as a next step we evaluated the migratory responses of BMMNCs isolated from caspase-1-KO mice to these chemoattractants by employing the Transwell migration system. First, as shown in Fig. 2A, we confirmed that BMMNCs from WT mice respond to eATP, S1P, and SDF-1 stimulation by activation of caspase-1. Next, Fig. 2B shows impaired migration of caspase-1-KO CFU-GM clonogenic progenitors in response to these major HSPC chemoattractants. Caspase-1-KO clonogenic progenitors also showed decreased chemotaxis in response to SDF-1 in the presence of cathelicidin (LL-37), which sensitizes the chemotactic responsiveness of HSPCs, as we demonstrated in the past [4, 24, 26].

**Fig. 3:**
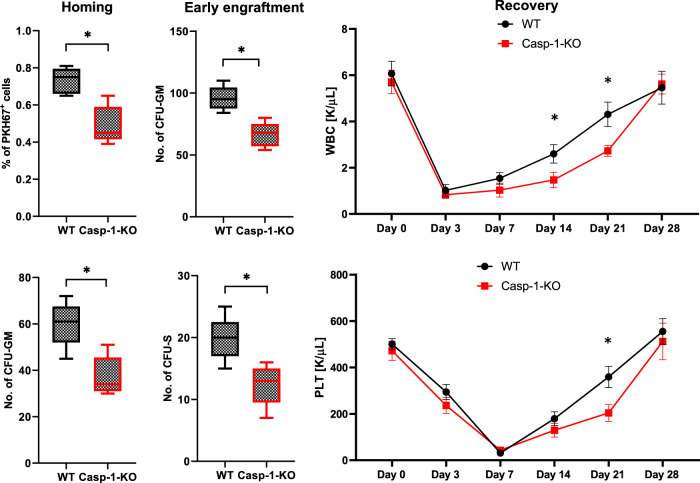
Defective homing and engraftment of caspase-1-KO BMMNCs in control animals. Left panels (early homing experiments): FACS analysis of PKH67 green fluorescent-labeled BM cells harvested from lethally irradiated and transplanted WT mice. These mice were transplanted (24 h previously) with PKH67-labeled WT or Casp1-KO mouse BM cells (top, left panel), and CFU-GM clonogenic progenitors (bottom, left panel) formed from the BM samples were enumerated as described in Materials and methods. Middle panels (early engraftment experiments): 12 days after transplantation (WT or Casp1-KO BM cells) into WT mice, BMMNCs or spleens from the transplanted mice were harvested and cultured for CFU-GM (top, middle panel) or CFU-S colonies (bottom, middle panel). No colonies were observed in lethally irradiated or normal control (untransplanted) mice. Right panels: lethally irradiated WT mice were transplanted with BMMNCs from WT or Casp1-KO mice, and peripheral blood samples were withdrawn for WBC (top, right panel) and platelet (PLT, bottom, right panel) analysis on days 0, 3, 7, 14, 21, and 28 after transplantation. The data are presented as means ± SE, and an unpaired Student’s *t*-test was used for determination of significance (*p ≤ 0.05).

To better address this defective migration of caspase-1-KO HSPCs, we moved to an in vivo model in which we transplanted control WT mice with BMMNCs isolated from caspase-1-KO mice and compared homing and engraftment of these cells with transplantations performed with BMMNCs isolated from WT animals (Fig. 3). We found that the number of donor-derived, PKH67-labeled BMMNCs and the number of CFU-GM clonogenic progenitors (as enumerated 24 h after transplantation in BM from recipient mice, in a similar manner as day-12 colony-forming units in spleen [CFU-S] and day-12 CFU-GM clonogenic progenitors in BM) was reduced when BMMNCs from caspase-1-KO mice were transplanted. In parallel, we also observed a slowing in the recovery kinetics of leukocytes and blood platelets. This result indicates that HSPC expression of caspase-1 is involved not only in vitro but also in in vivo migration of HSPCs in response to BM chemotactic factors and affects their homing and engraftment in BM.

### AMD3100- and G-CSF-induced expression of mRNAs for Nlrp3 inflammasome components and crucial DAMPs is impaired in BMMNCs from caspase-1-KO animals

**Fig. 4:**
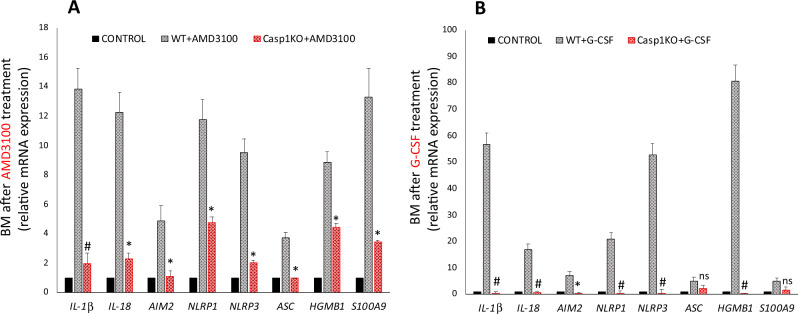
Caspase-1-KO BMMNCs weakly upregulate mRNAs for inflammasome genes in response to G-CSF- and AMD3100-induced mobilization. Left: normalized qRT-PCR expression analysis of *IL-1β, Il-18, Aim2, Nlrp1, Nlrp3, Asc (Pycard), Hmgb1, and S100A9* mRNAs from BMMNC samples obtained from WT or Casp1-KO mice injected with PBS or AMD3100 (5 mg/kg). Right: relative expression of *IL-1β, Il-18, Aim2, Nlrp1, Nlrp3, Asc (Pycard), Hmgb1, and S100A9* mRNAs in samples obtained from WT or Casp1-KO mice injected with PBS or G-CSF (180 μg/kg). The data represent the mean value ± SEM for three independent experiments. Results of qRT-PCR were normalized to the *β2-microglobulin* (*β2m*) expression levels. *p ≤ 0.05 and ^#^*p* ≤ 0.005.

To address the observed differences at the molecular level, we evaluated changes in expression of mRNAs for Nlrp3 inflammasome components and selected DAMPs in BMMNCs in response to mobilization with AMD3100 and G-CSF (Fig. 4). We found that, in comparison with WT animals, caspase-1-KO mice have significantly reduced expression of mRNAs encoding Nlrp3, IL-1β, IL-18, HMGB1, and S100A9 as well mRNAs for other inflammasomes, such as Aim2 and Nlrp1. This defective expression was particularly visible in BMMNCs from mice mobilized for 6 days with G-CSF.

### Neither IL-1β nor IL-18 mobilize HSPCs in caspase-1-KO or Nlrp3-KO animals, but mobilization in these murine strains is enhanced after administration of a DAMP cocktail

**Fig. 5:**
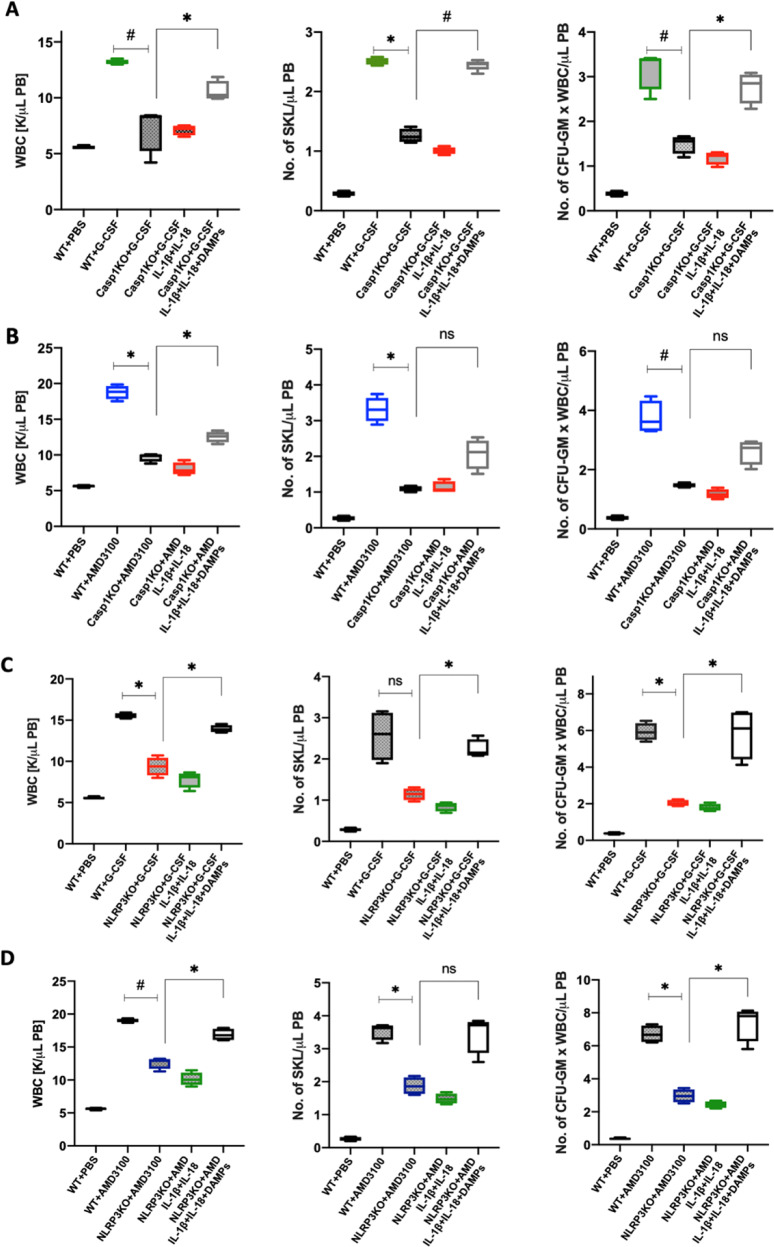
G-CSF and AMD3100 mobilization in caspase-1-KO and Nlrp3-KO animals is not restored by supplementation with IL-1β + IL-18 but requires the presence of DAMPs. **A** WBCs (left), SKL cells (middle), and CFU-GM clonogenic progenitors (right) were obtained from WT mice injected with PBS or G-CSF (180 µg/kg) or Casp1-KO mice injected with G-CSF with or without a mixture of IL-1β and IL-18 (0.5 µg each per mouse) or a DAMP cocktail (ATP [3 mg/kg] + HMGB1 [1.5 μg] + S100A9 [2 μg] per mouse). **B** WBCs (left), SKL cells (middle), and CFU-GM clonogenic progenitors (right) were obtained from WT mice injected with PBS or AMD3100 (5 mg/kg) or Casp1-KO mice injected with AMD3100 with or without a mixture of IL-1β and IL-18 (0.5 µg each per mouse) or a DAMP cocktail (ATP [3 mg/kg] + HMGB1 [1.5 μg] + S100A9 [2 μg] per mouse). AMD3100 was injected once, whereas G-CSF was administered on 4 consecutive days. AMD3100-injected mice were sacrificed 1 h post injection, whereas G-CSF-treated animals were sacrificed 6 h after the last injection. The number of SKL cells and CFU-GM progenitors mobilized into PB was calculated using the formula described in Materials and Methods. The data are presented as means ± SE, and an unpaired Student’s *t*-test was used for the determination of significance (*p ≤ 0.05 and ^#^*p* ≤ 0.005). **C** WBCs (left), SKL cells (middle), and CFU-GM clonogenic progenitors (right) were obtained from WT mice injected with PBS or G-CSF (5 mg/kg) or Nlrp3-KO mice injected with G-CSF with or without a mixture of IL-1β and IL-18 (0.5 µg each per mouse) or a DAMP cocktail (ATP [3 mg/kg] + HMGB1 [1.5 μg] + S100A9 [2 μg] per mouse). **D** WBCs (left), SKL cells (middle), and CFU-GM clonogenic progenitors (right) obtained from WT mice injected with PBS or AMD3100 (5 mg/kg) or Nlrp3-KO mice injected with AMD3100 (5 mg/kg) with or without a mixture of IL-1β and IL-18 (0.5 µg each per mouse) or a DAMP cocktail (ATP [3 mg/kg] + HMGB1 [1.5 μg] + S100A9 [2 μg] per mouse). AMD3100 was injected once, whereas G-CSF was administered on 4 consecutive days. AMD3100-injected mice were sacrificed 1 h post injection, whereas G-CSF-treated animals were sacrificed 6 h after the last injection. The numbers of SKL cells and CFU-GM progenitors mobilized into PB were calculated using the formula described in Materials and methods. The data are presented as means ± SE, and an unpaired Student’s *t*-test was used for the determination of significance (*p ≤ 0.05; ^#^*p* ≤ 0.005).

Next, to see whether co-administration of IL-1β and IL-18 would improve the poor mobilization status of caspase-1-KO animals, we mobilized these mice with G-CSF or AMD3100, alone or in the presence of injected IL-1β + IL-18, and expected to see improvement in mobilization efficacy. To our surprise, however, we did not observe any positive effect on mobilization (Fig. 5A, B). Based on this result, we performed a similar experiment in Nlrp3-KO mice, and again we did not observe improvement in mobilization (Fig. 5C, D) in this poorly mobilizing murine strain, as reported in our previous work [7, 9].

We reported in the past that in vivo injection of a DAMP cocktail (eATP + HGMB1 + S100A9) enhances G-CSF-induced mobilization of HSPCs and activation of the ComC [5, 16, 24, 33]. Since DAMPs are released from the cells in an Nlrp3 inflammasome–caspase-1-dependent manner, we employed this DAMP cocktail again to see whether we could enhance G-CSF- and AMD3100-induced mobilization in caspase-1-KO and Nlrp3 inflammasome-deficient, poorly mobilizing mice. We found that administration of this cocktail of DAMPs restored defective mobilization in caspase-1-KO animals (Fig. 5A, B), as in Nlrp3-KO mice (Fig. 5C, D).

**Fig. 6:**
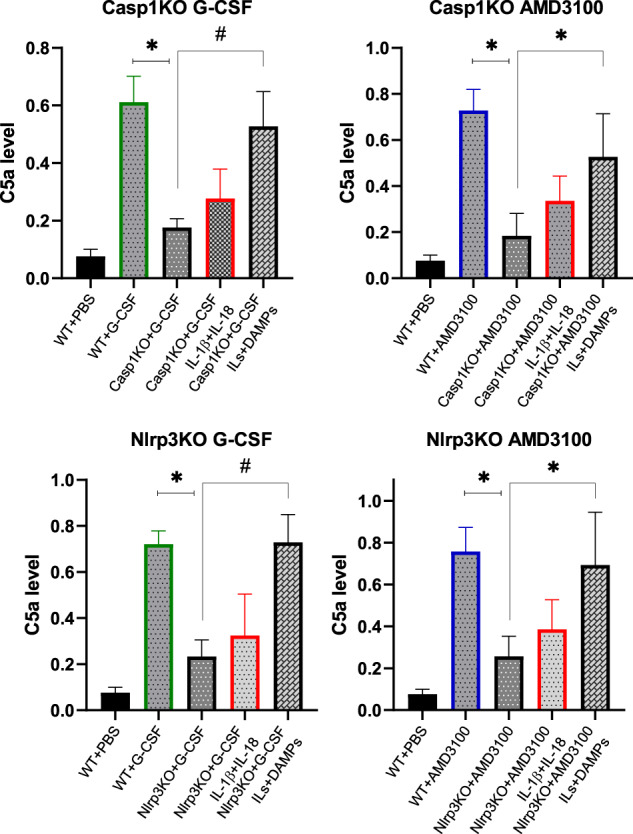
DAMPs activate the ComC in BMMNCs. The levels of C5a protein in conditioned media harvested from BMMNC from WT or Casp1-KO (upper panel) mobilized by G-CSF or AMD3100 and from WT or Nlrp3-KO BMMNCs (lower panel), in the presence or absence of IL-1β+IL-18 and in the presence of IL-1β + IL-18 + DAMPs (eATP, HMGB1, S1009A) were measured by ELISA. The data represent the mean value for two independent experiments (*p ≤ 0.05 and ^#^*p* ≤ 0.005).

DAMPs released into the extracellular space may activate the ComC, of which the distal part, involving the release of C5 cleavage fragments, is crucial for optimal egress of HSPCs from BM into PB [5, 16, 28, 36]. Figure 6 shows that the ComC is not efficiently mobilized in caspase-1-KO and Nlrp3-KO animals after administration of G-CSF or AMD3100, but its activation is significantly increased not by addition of IL-1β+IL-18 but in response to infused DAMPs. This is supported by performed Glow assay (Supplementary Fig. 3) to detect activated caspase-1 in Nlrp3-KO mice after administration of cocktail of DAMPs (eATP + HGMB1 + S100A9), and suggests involvement of other inflammasomes.

## Discussion

**Fig. 7:**
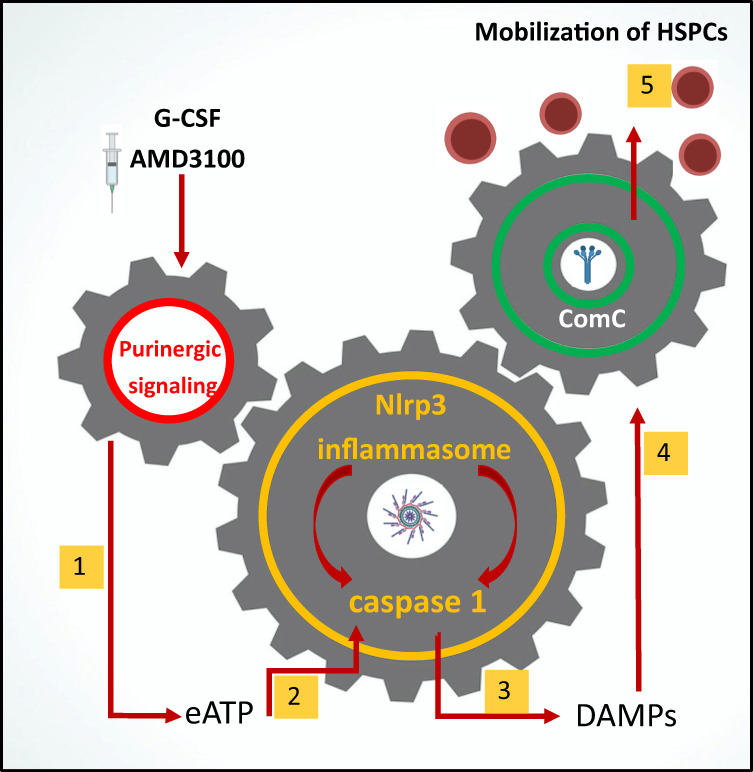
Nlrp3 inflammasome–caspase-1-mediated release of DAMPs as a gear mechanism that couples purinergic signaling with the complement cascade (ComC) during HSPC mobilization. Pro-mobilizing stimuli (e.g., G-CSF or AMD3100) (**1**) release ATP from activated innate immunity cells and HSPCs (**2**), which as extracellular ATP (eATP) activates intracellular inflammasomes via P2X4 and P2X7 purinergic receptors (**3**). As a result of Nlrp3 inflammasome and caspase-1 activation, several DAMPs are released, including HMGB1 and S100a9 (**4**), that activate the ComC. Activation of the ComC leads to the release of C5 cleavage fragments that are crucial as demonstrated in the past (refs 4, 28, and 46) for optimal release of HSPCs from BM into PB (**5**).

The most important outcome of this work is the discovery that the pro-mobilizing effects of the Nlrp3 inflammasome–caspase-1 axis depend on the release of DAMPs from hematopoietic cells. This process is maintained, on the one hand, by an autocrine positive-feedback mechanism involving Nlrp3 inflammasome activation, and, on the other hand, by activators of the ComC that are required for optimal egress of HSPCs from BM into PB. As depicted in Fig. 7, this result also sheds new light on the role of IL-1β and IL-18 in the mobilization of HSPCs, in which they stimulate hematopoietic cells to release DAMPs to activate the ComC. Moreover, we demonstrated that caspase-1-deficient HSPCs show a decrease in migration in response to BM homing factors and engraft more poorly after transplantation. Overall, these results also, for the first time, identify caspase-1 as an important orchestrator of HSPC trafficking.

There are several overlapping pathways involved in the egress of HSPCs from their niches into the circulation [37–44]. This process is initiated by pro-mobilizing cytokines (e.g., G-CSF and Gro-β), which are small molecular antagonists of receptors involved in the retention of HSPCs in BM niches (e.g., CXCXR4 and VLA-4), and by activation of signaling pathways involving mediators of purinergic signaling (eATP), adrenergic neurotransmitters, or bioactive phosphosphingolipids (S1P) [24, 26, 32–35, 45]. In parallel, evidence has accumulated that all of these factors that trigger the mobilization process induce a state of sterile inflammation in the BM microenvironment, which produces activation of the ComC [4, 5, 28, 46], release of proteolytic and lipolytic enzymes from cells [17, 40, 44, 47], and activation of the coagulation cascade [4, 46]. In parallel, several proinflammatory mediators are also released that maintain this state, as are other mediators that control this process [16–19, 21, 48].

Our recent research proposes that central roles in maintaining sterile inflammation in BM are played by the innate immunity response and activation of the Nlrp3 inflammasome, which becomes activated both in innate immunity cells and, in parallel, in HSPCs, as we demonstrated [7–9, 24, 26]. This wide-ranging expression of Nlrp3 inflammasomes in cells involved in the immune response and in HSPCs should not be surprising, as all these cells have a common developmental precursor. Thus, we propose that all mediators that trigger the mobilization process merge at the Nlrp3 inflammasome and initiate activation of caspase-1 and release of the proinflammatory cytokines IL-1β and IL-18 and several DAMPs from the cells.

Interestingly, IL-1β or IL-18 independently induce mobilization of HSPCs, as demonstrated in the past [7–9, 24], but they turned out to be ineffective in Nlrp3-KO and caspase-1-KO mice, as we report here. This finding indicates that their role depends on the presence of a functional Nlrp3 inflammasome–caspase-1 axis in hematopoietic cells. Therefore, if injected in vivo, both cytokines activate Nlrp3 inflammasomes in BM cells, or, if secreted in a caspase-1-dependent manner from Nlrp3 inflammasome-expressing cells, promote and maintain a state of sterile inflammation in BM by employing autocrine/paracrine positive-feedback loops.

We report that the poorly mobilizing state in Nlrp3-KO and caspase-1-KO mice was reversed after injection of a cocktail of the most relevant DAMPs (eATP + HGMB1 + S100A9), which are secreted from cells in an Nlrp3 inflammasome–caspase-1-dependent manner. This result indicates that a crucial role of this axis in response to pro-mobilizing cues is release of these mediators. We have already reported that DAMPs injected into WT animals activate the ComC, as evidenced by detection of the C5a cleavage fragment in PB [5, 16, 24, 28]. This finding is supported by other published observations that DAMPs may activate the ComC in several experimental settings of inflammation or tissue damage [18, 19, 49, 50].

Thus, our results demonstrate for the first time the crucial role of the Nlrp3 inflammasome–caspase-1 axis in the release of DAMPs, which activate the ComC to execute the mobilization process. At the molecular level we report that caspase-1-KO BMMNCs exposed to G-CSF and AMD3100 show reduced levels of mRNAs encoding protein components of the Nlrp3 inflammasome. These cells also have reduced levels of mRNAs for other inflammasomes, including mRNAs for Nlrp1 and Aim1. Thus, future research should focus on the potential involvement of other members of the inflammasome family in regulating the trafficking of HSPCs. For example, it has been proposed that Aim1 inflammasomes are also activated by DAMPs [36, 51]. More importantly, the poor mobilization status of caspase-1-KO and Nlrp3-KO mice is correlated with defective activation of the ComC, and mobilization ability is restored after injection of DAMPs.

Moreover, we observed in the current work that caspase-1 promotes migration of HSPCs in response to BM-expressed chemoattractants, such as the major chemoattractant SDF-1 [24, 26, 34], as well as other supportive homing factors, such as S1P and eATP [20, 24, 26, 32, 45]. By employing functional immunofluorescence-based enzymatic assays, we demonstrated activation of caspase-1 in BMMNCs in response to BM homing factors. The requirement for caspase-1 in the normal migration of HSPCs has been shown in Transwell migration assays and was finally verified in vivo by demonstrating that BMMNCs from caspase-1-KO mice have defective homing and engraftment in WT animals. These results are similar to those reported in Nlrp3-KO animals [7, 8]. To explain this outcome at the molecular level, Nlrp3 inflammasome activation plays an important role in promoting membrane lipid raft formation, which assembles the cell-surface-expressed CXCR4 homing receptor for SDF-1 with downstream signaling proteins and thus optimizes the chemotactic homing response [24, 26, 32, 34, 45]. An important enhancer of membrane lipid raft formation is eATP, which is one of the major DAMPs [20, 24, 26] in addition to some of the cleavage fragments of the activated ComC, such as the anaphylatoxin C3a [52]. Lipid raft formation is also stimulated by certain other mediators of innate immunity, such as cathelicidin (LL-37) [24, 26]. Therefore, release of DAMPs, including eATP, activation of the ComC, and involvement of other mediators of activated innate immunity (e.g., LL-37) play important roles in this phenomenon and are defective in mice that lack a functional Nlrp3 inflammasome–caspase-1 axis.

It is important to mention that our past and recent results indicate the presence of an intrinsic auto-navigation mechanism in HSPCs that regulates their egress from BM as well as migration after transplantation to BM niches, which is modulated, positively or negatively, by external cues. Our current results support a central role for the Nlrp3 inflammasome–caspase-1 axis. This auto-navigation mechanism is activated in cells in response to HSPC chemoattractants and negatively regulated by heme oxygenase 1 [53], inducible nitric oxide synthetase [27], and endogenous adenosine [26, 54], which are known negative regulators of Nlrp3 inflammasome activation. As would promoting activation of the Nlrp3–caspase-1 axis, inhibition of these negative regulators of stem cell trafficking would improve the efficacy of this process.

In conclusion, we have provided further evidence that the Nlrp3 inflammasome–caspase-1 axis promotes pharmacological mobilization, migration, and homing of HSPCs by (i) release of DAMPs that activate the intrinsic navigation mechanism in response to major BM chemoattractants in HSPCs, and (ii) promoting and maintaining a state of sterile inflammation in the BM microenvironment and activation of the ComC. Thus, these results support important roles for innate immunity, BM sterile inflammation in response to pro-mobilizing agents, and involvement of the Nlrp3 inflammasome and ComC in the trafficking of HSPCs and shed more light on the DAMPs that modulate [4, 5, 21, 26, 39] these processes.

## Supplementary information

Figure Legends

Supplementary Figure 1

Supplementary Firgure 2

Supplementary Figure 3
