# Supplementary material for: Danger-associated molecular pattern molecules take unexpectedly a central stage in Nlrp3 inflammasome–caspase-1-mediated trafficking of hematopoietic stem/progenitor cells
Source: Leukemia. 2021 Feb 23;35(9):2658–71. doi: 10.1038/s41375-021-01158-9 (PMC8410600; doi:10.1038/s41375-021-01158-9)
Supplement: Supplementary file 1 — Figure Legends [file 41375_2021_1158_MOESM1_ESM.docx]

**Supplementary Figure Legends**.

**Supplementary Figure 1. Inhibition of caspase-1 in wild type animals results in poor G-CSF and AMD3100 mobilization**. **A**, representative data show the PB parameters for WBCs (**left**), SKL cells (**middle**), and CFU-GM clonogenic progenitors (**right**) in samples obtained from WT mice injected with PBS or G-CSF (180 µg/kg), without or with the caspase-1 inhibitor VX765 (40 mg/kg). **B**, representative results for the PB parameters for WBCs (**left**), SKL cells (**middle**), and CFU-GM clonogenic progenitors (**right**) from WT mice injected with PBS or AMD3100 (5 mg/kg), without or with the caspase-1 inhibitor VX765 (40 mg/kg). AMD3100 was injected once, and mice were sacrificed 1 h post-injection, whereas the G-CSF was injected daily for 4 days, and the mice were sacrificed 6 h after the last injection. The numbers of SKL and progenitor cells mobilized into PB were calculated using the formula, WBCs x SKL cells/gated WBCs = SKL cells/μl; and CFU-GM/μl of PB was evaluated by the formula, [WBCs] x CFU-GM colonies/WBCs plated. The data are presented as means ± S.E., and an unpaired Student’s t-test was used for the determination of significance (**p* ≤ 0.05, ^#^*p* ≤ 0.005 and ^##^*p* ≤ 0.005 WT+PBS *vs* WT+G-CSF+VX765).

**Supplementary Figure 2. The number of clonogenic progenitors and SKL cells in caspase-1-KO mice.** PB parameters for SKL cells (**left**), CFU-GM clonogenic progenitors (**middle**), and BFU-E clonogenic progenitors (**right**) obtained from BMMNC samples from WT or Casp1-KO mice are shown. BMMNCs were stained with appropriate antibodies (see Materials and Methods), and SKL cells were analyzed by FACS. BMMNCs were cultured on human methylcellulose semi-solid medium supplemented with appropriate cytokines, and CFU-GM and BFU-E colonies were counted using a simple inverted microscope, as mentioned in Materials and Methods. The data are presented as means ± S.E., and an unpaired Student’s t-test was used for the determination of significance (**p* ≤ 0.05).

**Supplementary Figure 3.** Results of Glow assay to assess activation of caspase 1 in BMMNC from normal and Nlrp3-KO mice stimulated by cocktail of DAMPs.
