# Supplementary figures and images for: Danger-associated molecular pattern molecules take unexpectedly a central stage in Nlrp3 inflammasome–caspase-1-mediated trafficking of hematopoietic stem/progenitor cells
Source: Leukemia. 2021 Feb 23;35(9):2658–71. doi: 10.1038/s41375-021-01158-9 (PMC8410600; doi:10.1038/s41375-021-01158-9)

## Slide 1
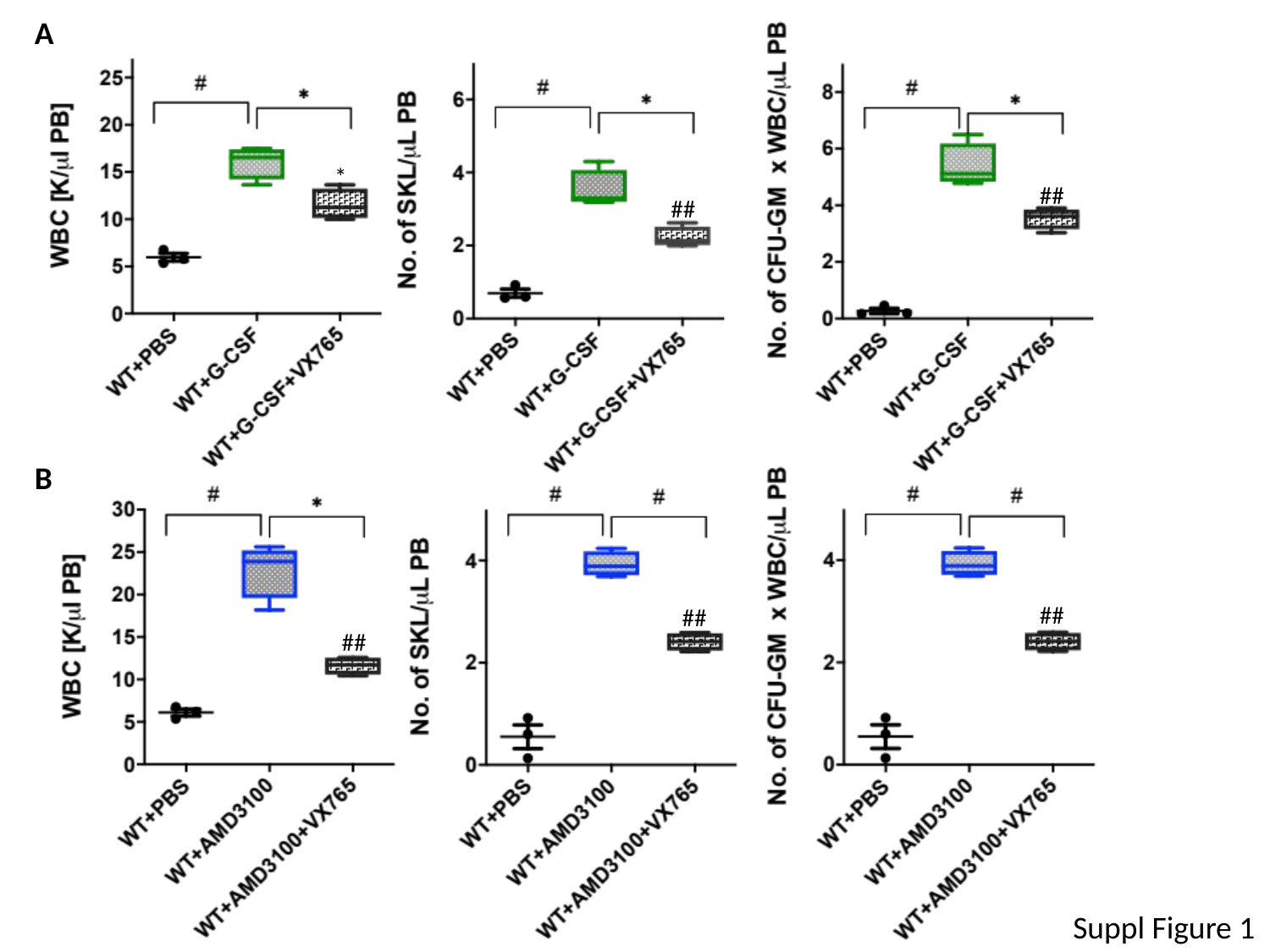

A
*
##
##
B
##
##
##
Suppl Figure 1

Supplement: Supplementary file 2 — Supplementary Figure 1 [file 41375_2021_1158_MOESM2_ESM.pptx]

## Slide 1
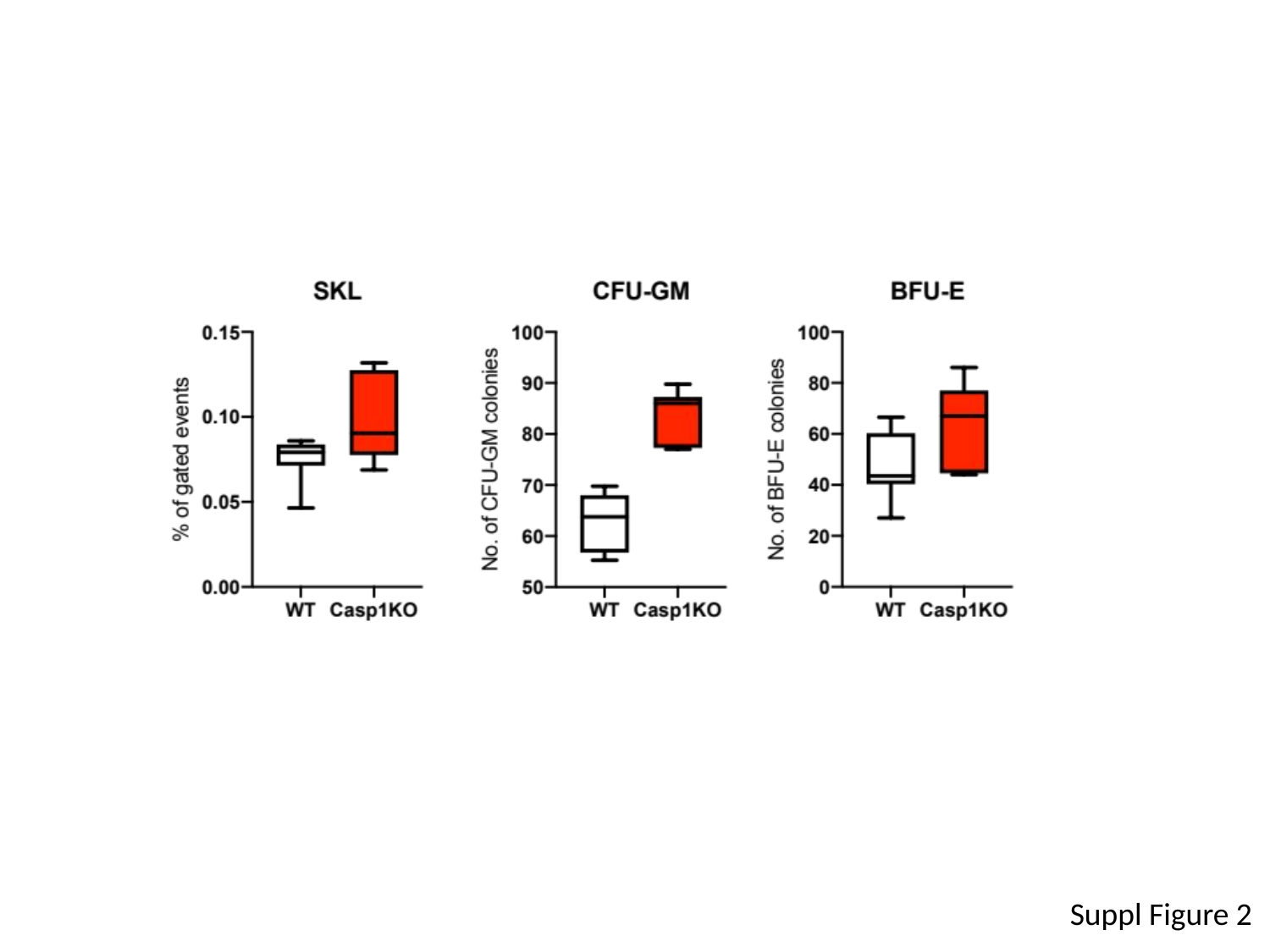

Suppl Figure 2

Supplement: Supplementary file 3 — Supplementary Firgure 2 [file 41375_2021_1158_MOESM3_ESM.pptx]
