# Supplementary material for: Danger-associated molecular pattern molecules take unexpectedly a central stage in Nlrp3 inflammasome–caspase-1-mediated trafficking of hematopoietic stem/progenitor cells
Source: Leukemia. 2021 Feb 23;35(9):2658–71. doi: 10.1038/s41375-021-01158-9 (PMC8410600; doi:10.1038/s41375-021-01158-9)
Supplement: Supplementary file 4 — Supplementary Figure 3 [file 41375_2021_1158_MOESM4_ESM.pptx]

## Slide 1
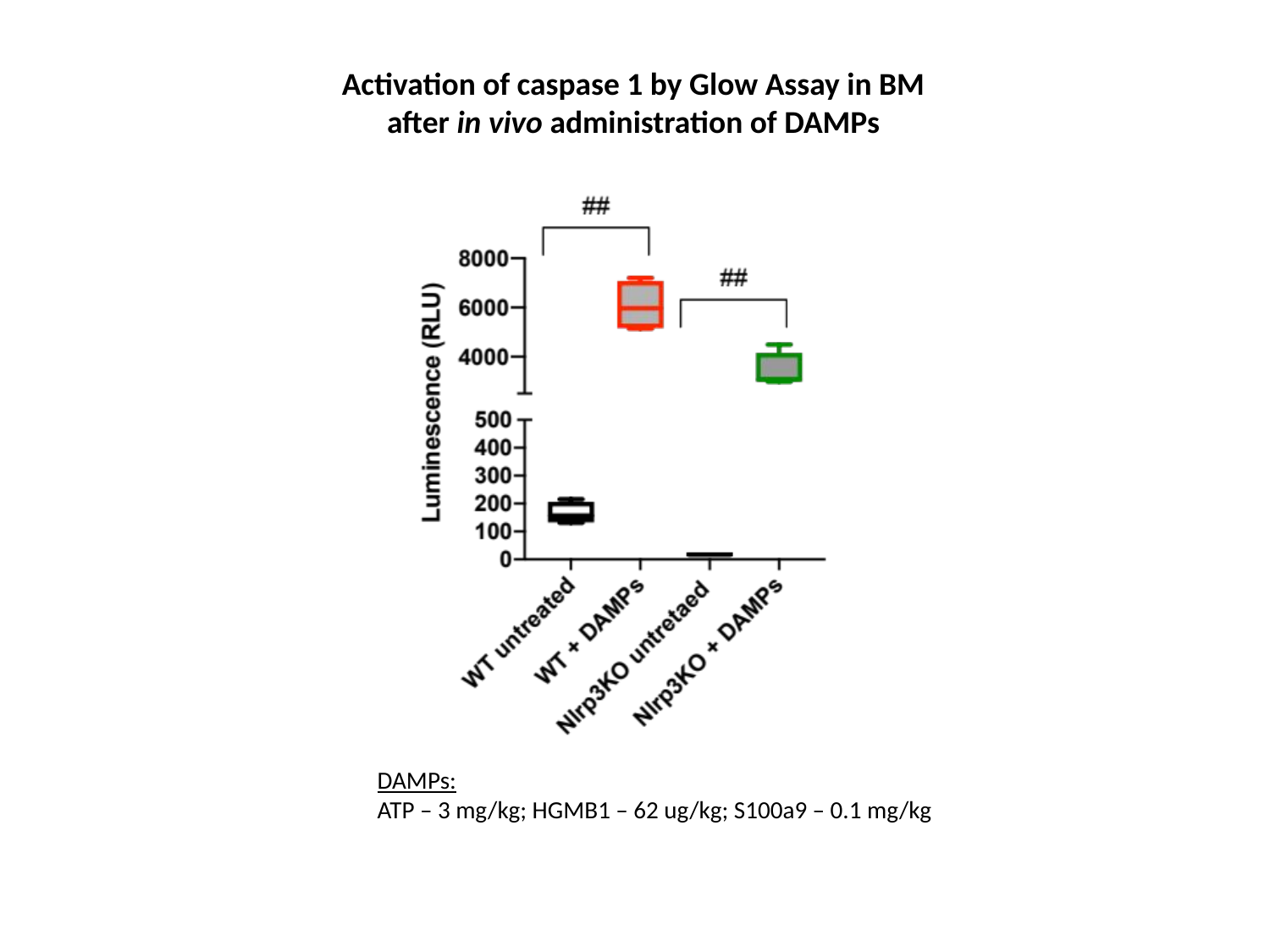

Activation of caspase 1 by Glow Assay in BM
after in vivo administration of DAMPs
DAMPs:
ATP – 3 mg/kg; HGMB1 – 62 ug/kg; S100a9 – 0.1 mg/kg
